# Improving the estimation of the death rate of infected cells from time course data during the acute phase of virus infections: application to acute HIV-1 infection in a humanized mouse model

**DOI:** 10.1186/1742-4682-11-22

**Published:** 2014-05-21

**Authors:** Hiroki Ikeda, Rob J de Boer, Kei Sato, Satoru Morita, Naoko Misawa, Yoshio Koyanagi, Kazuyuki Aihara, Shingo Iwami

**Affiliations:** 1 Department of Biology, Faculty of Sciences, Kyushu University, 6-10-1 Hakozaki, Higashi-ku, Fukuoka, Fukuoka, Japan; 2 Theoretical Biology & Bioinformatics, Utrecht University, Utrecht 3584 CH, the Netherlands; 3 Laboratory of Viral Pathogenesis, Institute for Virus Research, Kyoto University, Kyoto, Japan; 4 Department of Mathematical and Systems Engineering, Shizuoka University, Hamamatsu, Shizuoka 432-8561, Japan; 5 Institute of Industrial Science, The University of Tokyo, Meguro-ku, Tokyo, Japan; 6 Graduate School of Information Science and Technology, The University of Tokyo, Bunkyo-ku, Tokyo, Japan; 7 Precursory Research for Embryonic Science and Technology (PRESTO), Japan Science and Technology Agency (JST), Kawaguchi, Saitama, Japan

**Keywords:** Population dynamics model, Parameter estimation, Virus infection dynamics, Death rate of infected cells, HIV-1, Humanized mouse model

## Abstract

**Background:**

Mathematical modeling of virus dynamics has provided quantitative insights into viral infections such as influenza, the simian immunodeficiency virus/human immunodeficiency virus, hepatitis B, and hepatitis C. Through modeling, we can estimate the half-life of infected cells, the exponential growth rate, and the basic reproduction number (*R*_0_). To calculate *R*_0_ from virus load data, the death rate of productively infected cells is required. This can be readily estimated from treatment data collected during the chronic phase, but is difficult to determine from acute infection data. Here, we propose two new models that can reliably estimate the average life span of infected cells from acute-phase data, and apply both methods to experimental data from humanized mice infected with HIV-1.

**Methods:**

Both new models, called as the reduced quasi-steady state (RQS) model and the piece-wise regression (PWR) model, are derived by simplification of a standard model for the acute-phase dynamics of target cells, viruses and infected cells. By having only a limited number of parameters, both models allow us to reliably estimate the death rate of productively infected cells. Simulated datasets with plausible parameter values are generated with the standard model to compare the performance of the new models with that of the major previous model (i.e., the simple exponential model). Finally, we fit models to time course data from HIV-1 infected humanized mice to estimate the several important parameters characterizing their acute infection.

**Results and conclusions:**

The new models provided much better estimates than the previous model because they more precisely capture the *de novo* infection process. Both models describe the acute phase of HIV-1 infected humanized mice reasonably well, and we estimated an average death rate of infected cells of 0.61 and 0.61, an average exponential growth rate of 0.69 and 0.76, and an average basic reproduction number of 2.30 and 2.38 in the RQS model and the PWR model, respectively. These estimates are fairly close to those obtained in humans.

## Background

In most viral infections, the initial exponential growth phase is followed by a second exponential phase known as contraction. In hosts exposed to viruses such as influenza and coronaviruses (the causative agents of severe acute respiratory syndrome), the viral load continuously declines during the contraction phase [1,2]. In contrast, in chronic viral infections, such as human immunodeficiency virus (HIV) and hepatitis C virus (HCV) infections, contraction slows down such that the viral load approaches a steady state, called the virological set point [3,4]. In both infection types, the expansion and contraction of the viral load have been modeled as single exponential functions, with parameters determined by linear regression of the log transformed data [3,5-7]. This simple approach is reasonable as long as the conditions, e.g., the availability of target cells or the immune response, hardly change within each phase. Using this approach, the initial growth rate, death rate of the infected cells, and the basic reproduction number (i.e., *R*_0_) have been estimated, which has improved our understanding of particular virus infections, and has guided medical treatment [7-9]. For example, once the basic reproduction number is estimated, the critical inhibition, 1–1/*R*_0_, induced by vaccines, or by antiviral drugs, to prevent primary virus infection can be calculated [6].

Knowledge of the death rate of infected cells is crucial for properly understanding viral dynamics because the average life time is required for calculating the basic reproduction number. In chronic viral infections such as HIV and HCV, the death rate is estimated from large perturbations of the set point viral load data instigated by potent anti-viral therapy [4,10-13]. Shortly after effective treatment, the decay rate of viral load approaches the death rate of productively infected cells. However, estimating the death rate of infected cells during the acute phase remains a challenging task, and, as a consequence, it is difficult to accurately estimate the basic reproduction number from viral load data during the early stages of viral infection. In addition to calculation of the basic reproduction number, the death rate *per se* is useful for evaluating the efficacy of vaccine-induced cellular immune responses during the acute phase of virus infection [14-16]. Therefore, an improved method for estimating the death rate of infected cells during this phase is urgently required.

In this study, we first generated simulated datasets with biologically plausible parameter values, using a population dynamics model of virus population. The time evolution of target cell densities and viral load were modeled during the acute phase. The datasets describing acute infection were subsequently analyzed by two novel mathematical models to evaluate whether the new models could accurately estimate the known parameters. Our proposed models properly described the artificial datasets and delivered better estimates of the parameters and well calculated indices than conventional models (i.e., simple exponential models). Our methods proved especially effective for calculating the death rate of infected cells. We then applied our models to time course data from a human hepatopoietic stem cell-transplanted humanized mouse model infected with HIV type-1 (HIV-1) [17-20], to quantify the infection dynamics during the acute phase. To our knowledge, this is the first report quantifying the dynamics of acute HIV-1 infection in humanized mice. Finally, we discuss how our approach may be combined with animal experiments. Like previous simple exponential models [3,5-7], our approach is quite general and can be used in several infection models.

## Methods

### Mathematical models describing the acute phase of virus infection

#### ***(I) Reduced quasi-steady state (RQS) model***

The standard model for viral infection consists of three differential equations for target cells, *T*(*t*), infected cells, *I*(*t*), and viral particles, *V*(*t*) [7-9]. Since during acute infection the normal production and loss of target cells is much smaller than the loss due to viral infection and/or its side effects [14-16], the standard model can be reduced to

(1) $$T'\left(t\right)=-\beta T\left(t\right)V\left(t\right),$$

(2) $$I'\left(t\right)=\beta T\left(t\right)V\left(t\right)-\delta I\left(t\right),$$

(3) $$V'\left(t\right)=\mathrm{pI}\left(t\right)-\mathrm{cV}\left(t\right),$$

where the parameters *β*, *δ*, *p* and *c* represent the conventional rate constants for viral infection of target cells, the death rate of infected cells, the virus production rate in an infected cell, and the clearance rate of virus particles, respectively. The initial expansion of viral load in this model is well approximated by *V*(*t*) ≈ *V*(0) exp (*g*_0_*t*) [3,5-7], with an exponential growth rate, *g*_0_ (the Malthusian parameter), given by the positive root of the characteristic equation *g*_0_^2^ + (*c* + *δ*)*g*_0_ + *cδ* - *pβT*(0) = 0, i.e., $g_{0}=\left\{-\left(c+\delta \right)+\sqrt{{\left(c-\delta \right)}^{2}+4p\beta T\left(0\right)}\right\}/2$.

This model can be simplified further by a quasi-steady state (QSS) approximation for the viral particles [7,21-23]. Typical estimated half-lives of viruses (1/*c*) such as HIV, HCV and hepatitis B virus are of the order of minutes (or hours), whereas those of infected cells (1/δ) *in vivo* are of the order of days [4,5,7-13]. Since, the clearance rate of viral particles, *c*, is typically much larger than the death rate, *δ*, of the infected cells, we can make a QSS assumption, *V*′(*t*) = 0, and replace Eq. (3) by *V*(*t*) = *pI*(*t*)/*c*. Because we fit viral loads, *V*(*t*), rather than the number of infected cells, *I*(*t*), we also substitute *I*(*t*) = *cV*(*t*)/*p* into Eq. (2) to obtain

(4) $$V'\left(t\right)=\mathrm{rT}\left(t\right)V\left(t\right)-\delta V\left(t\right),$$

where *r* = *pβ*/*c* is the viral replication rate per target cell, and *δ* is the death rate of infected cells. Eqs. (1) and (4) together form our first model, that we here call the “reduced quasi-steady state” (RQS) model. The RQS model lumps the 8 parameters of the reduced standard model of Eqs. (1–3) into five parameters, i.e., *β, r, δ, T*(*0*), and *V*(*0*). Because there is no production of target cells the infection will ultimately be cleared. The five parameters together define several “observables”. First, the basic reproduction number is *R*_0_ = *rT*(0)/*δ*. Second, the initial exponential growth rate is *g*_0_ ≈ *rT*(0) - *δ* ( when *δ* and *g*_0_ ≪ *c*, one can directly calculate the same *g*_0_ from the characteristic equation). Third, the final level of target cells is given by the epidemiological “final size equation” [24,25], as the solution of *f* = exp [ - *R*_0_(1 - *f*)], where *f* = *T*_*min*_/*T*(0) is the fraction of surviving target cells.

#### ***(II) Piece wise targets (PWT) model***

**Figure 1:**
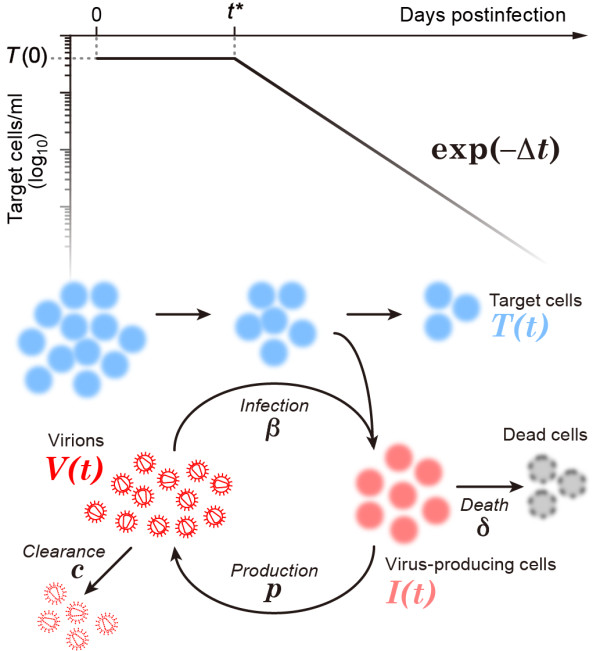
**Schematic of the novel exponential model.** The variables *T*(*t*), I(*t*) and *V*(*t*) denote the number of target and infected cells per ml and the amount of viruses per ml, respectively, at time *t*. The parameter Δ denotes the loss rate of target cells per day after the flat phase, *β* the rate constant for viral infection of target cells, and *c* the clearance rate of virus particles. *δ* denotes the death rate and *p* the viral production rate of infected cells. t^*^ represents the time at which the target cells start declining with slope –∆.

In the standard model, and its simplifications outlined above, the dynamics of the target cells are coupled to the density of viral particles because target cells disappear by infection. Since target cell densities in the peripheral blood (PB) may also depend on other factors, like inflammation, activation and redistribution, we next write a model where the target cell dynamics are decoupled from the viral dynamics. Since, during the acute phase of several virus infections, such as HIV, SIV and SHIV, the decrease in number of target cells in PB is preceded by an initial flat phase [14-20,26-28], we propose a phenomenological model for the target cells consisting of an initial flat phase, and a second phase of exponential loss (Figure 1). This basically implies that we assume that target cells only become a limiting factor when their density starts to decline. Thus, the dynamics of target cells is described as follows:

(5) $$\begin{array}{ccc} T\left(t\right)=T\left(0\right), & \mathrm{for} & t<t^{*}, \end{array}$$

(6) $$\begin{array}{ccc} T\left(t\right)=T\left(0\right)\text{exp}\left\{-\Delta \left(t-t^{*}\right)\right\}, & \mathrm{for} & t>t^{*}, \end{array}$$

where the parameter Δ represents the daily rate of target cells loss following the initial flat phase, and t^*^ corresponds to the time at which the target cell densities begin to decrease. Eqs. (4–6) define our second model that we here call the “piece wise targets” (PWT) model. The PWT model has six parameters, i.e., *β, r, δ, T*(*0*), *V*(*0*), Δ, and t^*^, and because it shares Eq. (4) with the RQS model, it has the same definitions for the replication rate, *r* = *pβ*/*c*, the Malthusian parameter, *g*_0_ = *rT*(0) - *δ*, and the reproduction number *R*_0_ = *rT*(0)/*δ*. In contrast to the partial depletion, *f*, in the RQS model, the target cells will ultimately be completely depleted, i.e., *T*(∞) → 0, in the PWT model. Thanks to the decoupled dynamics of the target cells the PWT model can be solved analytically:

(7) $$\begin{array}{ccc} V\left(t\right)=V\left(0\right)\text{exp}\left(g_{0}t\right), & \text{for} & t<t^{*}, \end{array}$$

(8) $$\begin{array}{ccc} V\left(t\right)=V\left(t^{*}\right)\text{exp}\left\{\frac{\mathrm{rT}\left(0\right)\left[1-\text{exp}\left(-\Delta \left(t-t^{*}\right)\right)\right]}{\Delta }-\delta \left(t-t^{*}\right)\right\}, & \text{for} & t>t^{*}, \end{array}$$

where the replication phase (Eq. (7)) is identical to the initial phase of the standard model (see the remark above). Thus, at the price of one additional parameter, we can generalize the depletion of target cells to mechanisms (e.g., inflammation, activation and redistribution of target cells) other than infection only, and have a model with very similar parameters characterizing the acute viral infection.

#### ***(III) Piece wise regression (PWR) model***

As a control method, we additionally consider a classical method that has been widely adopted in earlier literatures [3,5-7]. Previously acute infection data have been quantified using piece wise linear regression of the log transformed viral loads before and after the peak in the viral load [3,5-7]. The ascending and descending slopes of the log viral load roughly correspond to the exponential growth rate, *g*_0_, and the death rate, *δ*, of infected cells, respectively [3,5-7]. Since *g*_0_ = *rT*(0) - *δ* and *R*_0_ = *rT*(0)/*δ*, these two slopes suffice to estimate the basic reproduction number, *R*_0_ ≈ 1 + *g*_0_/*δ*, and knowing the initial target cell density, *T*(0), the viral replication rate per target cell can be estimated from the *R*_0_, i.e., *r* = *δR*_0_/*T*(0) [3,5-7]. Here we call this classical model as the “piece wise regression” model (PWR). It has been realized before that the down-slope will only reflect the death rate of infected cells, *δ*, if there is hardly any residual infection of target cells during the contraction phase, i.e., if target cells are markedly depleted [3,5-7].

### Artificial data generated from a population dynamics model of virus infection

To estimate the accuracy of the parameters estimated by our two novel models, we created simulated time course data of target cell densities and viral load during the acute phase of viral infection (lasting approximately 21 days [14-20,26-28]) assuming biologically plausible parameter values. The artificial datasets were generated with the reduced standard population dynamics model of viral infection, i.e., Eqs. (1–3), in which the target cell dynamics are coupled to the viral dynamics by the infection term.

We added stochastic variation to the “data” generated by this model by adding “observational” noise and/or by varying the parameter values. The log transformed data were perturbed by adding a normally distributed noise variable with zero mean and standard deviation *σ* (see Results). The datasets describing acute infection were subsequently analyzed by the two novel RQS and PWT models, and by the previous PWR model.

### HIV-1 infection in humanized mice

The dynamics of HIV-1 infection during acute infection were quantified in a human hepatopoietic stem cell-transplanted humanized mouse model (NOG-hCD34 mice) [17-20]. Five humanized mice were infected with the CCR5-tropic HIV-1 (strain AD8) [29], and 100 μl of peripheral blood (PB) was routinely collected under anesthesia through the retro-orbital venous plexus at 0, 3, 7, 14, and 21 days post-infection, as previously described [17-20]. The amount of viral RNA in 50 μl of plasma was quantified by RT-PCR (Bio Medical Laboratories, Inc). To estimate target cell densities, the number of memory CD4^+^ T cells was measured by hematometry and flow cytometry, as previously described [17-20]. Briefly, the number of human leukocytes in 10 μl of peripheral blood (PB) was measured in a Celltac α MEK-6450 hematology analyzer (Nihon Kohden, Co.), and the percentage of memory CD4^+^ T cells in human CD45^+^ leukocytes (i.e., CD45^+^ CD3^+^ CD4^+^ CD45RA^-^ cells) was quantified in a FACSCanto II (BD Biosciences) flow cytometer. In the flow cytometry analyses, APC-conjugated anti-CD4 antibody (RPA-4; Biolegend), APC-Cy7-conjugated anti-CD3 antibody (HIT3a; Biolegend), and PE-conjugated anti-CD45 antibody (HI30; Biolegend) were used.

All protocols involving human subjects were reviewed and approved by the Kyoto University institutional review board. Informed written consent from the human subjects was obtained in this study.

## Results

### Coverage probability of the mathematical models

In the Methods section we formulate two novel mathematical models describing the target cell densities and the viral load during acute infection. We created artificial data with target cell densities and virus loads during acute infection using the reduced standard model for viral infection (i.e., Eqs. (1–3)). The data was generated for one ml of PB with “typical” values of the parameters for HIV-1, i.e., an infection rate *β* = 10^-8^per cell per day, a virus production rate *p* = 4000 particles per day, a death rate of infected cells *δ* = 0.7 per day, and a clearance rate of *c* = 23 per day [7-11]. We study whether our simplified models can describe the *in silico* data, and whether their (lumped) parameters are identifiable. The major biological observables of this model are the initial viral growth rate, $g_{0}=\left\{-\left(c+\delta \right)+\sqrt{{\left(c-\delta \right)}^{2}+4p\beta T\left(0\right)}\right\}/2$, the viral replication rate per target cell, *r* = *pβ*/*c*, the death rate of infected cells, *δ*, and the basic reproduction number, *R*_0_ = *rT*(0)/*δ*.

**Figure 2:**
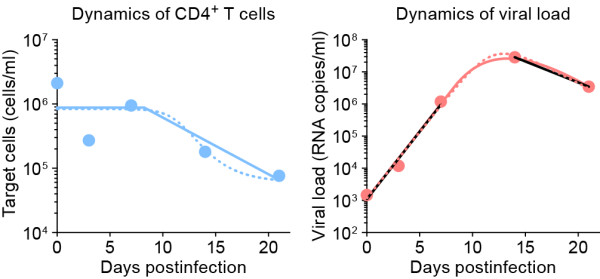
**Fitting of artificial (simulated) data by the novel models.** Artificial data were generated with the “reduced standard” viral infection model of Eqs. (1–3). The novel RQS model (Eqs. (1) and (4)), the novel PWT model (Eqs. (5–8)), and the previous PWR model were fitted to a total of 5 + 5 = 10 data points generated from the numerical solution of the reduced standard model at the days. Noise was added by perturbing the predicted log transformed value by a normally distributed error (with standard deviation *σ* = 0.2). The blue and red symbols denote one representative simulated dataset, the dotted line the best fit of the RQS model, the dashed line that of the PWT model, and the black solid lines show the ascending and descending slopes obtained with the PWR model. The true parameters were: *β* = 10^–8^, *p =* 4000, *δ* = 0.7, c = 23 and the initial values were *T*(0) = 1000000, *I*(0) = 0, and *V*(0) = 1000, corresponding an *R*_0_ = 2.48 and 90% target cell depletion (i.e., *f* = 0.1).

First, we created *in silico* data with “observational” noise by adding proportional random variation to each data point. Specifically, we drew random values from a Gaussian distribution with a mean of one and a standard deviation *σ* = 0.2, and added these values to the log transformed data. This seemed natural as we are also fitting the log transformed data, and on a log scale this corresponds to the measurement error of about 60%. The generated datasets were fitted to the numerical solutions of the RQS model (Eqs. (1) and (4)), the analytical solution of the PWT model (Eqs. (5–8)), and to the previous PWR model. The sum of squared residuals was minimized using the FindMinimum package of *Mathematica 9.0*, fitting the target cell and viral load data simultaneously. The typical behavior of the models using these best-fit parameter estimates is depicted in Figure 2, together with the simulated data. Other standard deviations of the parameters yielded similar results (results not shown). The two novel models reasonably describe the acute phase of viral infection. Note that target cell densities are partially depleted in the RQS model, and will ultimately approach “0” in the PWT model.

Second, we created different “patients” by randomly drawing the parameter values from normal distributions centered at their typical values. Thus, the infection rate, death rate of infected cells and virus production rate were assumed to be normally distributed as $\begin{array}{cc} \beta \sim N\left(\mu_{\beta },\sigma_{\beta }^{2}\right), & \delta \sim N\left(\mu_{\delta },\sigma_{\delta }^{2}\right), \end{array}$ and $p\sim N\left(\mu_{p},\sigma_{p}^{2}\right),$ with $\begin{array}{ccc} \mu_{\beta }=10^{-8}, & \mu_{\delta }=0.7, & \mu_{p}=4000, \end{array}$ respectively. The standard deviations were set as $\begin{array}{ccc} \sigma_{\beta }=10^{-9}, & \sigma_{\delta }=0.3, & \sigma_{p}=400 \end{array}$. In this way, we obtained a distribution of the basic reproduction number centered around the true value (*R*_0_ = 2.48). We then randomly sampled one parameter set of *β*, *δ*, *p* from the distributions, and produced 200 different artificial datasets as explained above. Analyzing each dataset with the same three models (RQS, PWT and PWR), we calculated 95% confidence intervals (CI) for *g*_0_, *r*, *δ* and *R*_0_, and investigated whether the 95% CI successfully contained the true values of *g*_0_, *r*, *δ* and *R*_0_ used to create the data set. This procedure was repeated for all 200 datasets, and Table 1 provides the frequency of datasets for which the 95% CI successfully contained the true values of *g*_0_, *r*, *δ* and *R*_0_, i.e., the coverage probability. Although the initial growth rate was well estimated by the previous PWR model, the novel RQS and PWT models estimated the viral replication rate, death rate of infected cells, and basic reproduction number much more accurately than the PWR model. Thus, the novel models can more accurately extract information from acute-phase viral infection data.

**Table 1:** The coverage probability of the PWR model, the RQS model, and the PWT model

**Method**	***r *** **(viral replication rate)**	***g***_**0 **_**(initial growth rate)**	***δ *** **(death rate)**	***R***_**0 **_**(basic reproduction number)**
Linear regression with the PWR model	0.25	0.87	0.12	0.25
Nonlinear fitting with the RQS model	0.86	0.83	0.72	0.78
Nonlinear fitting with the PWT model	0.31	0.58	0.19	0.33

### Sensitivity of parameter estimation to target cell depletion

At the beginning of the infection all models are identical as they all predict exponential growth of the virus load. The models differ around the peak because the previous PWR model assumes an exponential contraction after the peak, whereas the new models allow the peak be formed by the loss of target cells (i.e., by the *βT*(*t*)*V*(*t*) term in the RQS model, and by the exponential loss of target cells in the PWT model). In both models this loss of target cells continues during the contraction phase. However, if target cells were depleted rapidly such that there would be hardly any infection of target cells during the contraction phase, this difference among the models would vanish, and the contraction phase of the new models would also be dominated by the death rate of infected cells.

**Figure 3:**
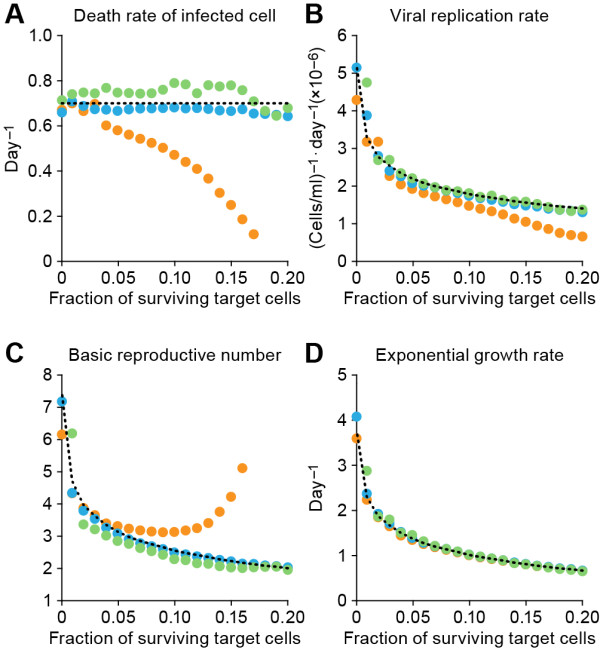
**Sensitivity of parameter estimates on target cell depletion, determined by our novel model.** For varying values of surviving target cell fraction (0.1%–20%), noiseless artificial datasets were generated, and fitted by our novel models and the simple exponential model to calculate **(A)** the death rate of infected cells, **(B)** viral replication rate, **(C)** the basic reproduction number, and **(D)** the exponential growth rate. The parameters are those used in Figure 2; only the infection rate *β* was altered to adjust the target cell depletion. When the other parameters were altered, similar results were obtained (data not shown). The blue, green and orange symbols plot the indices estimated by the RQS, PWT and PWR models, respectively. The black dotted lines depict the true parameter values.

The level to which the target cells become depleted in the reduced standard model of viral infection (Eqs. (1–3)) can be computed with the epidemiological “final size equation” [24,25] (see the Methods section). Using the same equation, Petravic *et al.*[16] show that this final size of the target cell level provides a good description of the nadir, *T*_*min*_, of the target cell density during an acute infection. Defining *f* = *T*_*min*_/*T*(0) as the fraction of surviving target cells, we use the final size equation to compute different values of the infection rate *β* to vary the nadir of the target cells over the interval *f* ∈ [0.001, 0.200]. Doing so we again created different cases, each with a different level of target cell depletion. For simplicity this was done in the absence of noise (which does not affect these results). Using the same approach as explained above we fit the “data” generated by these cases with the 3 models (Figure 3).

As discussed previously [3,5-7], the PWR model (orange symbols) fails to correctly estimate the death rate of infected cells in the presence of continued *de novo* infections, i.e., when many target cells survive; see Figure 3A (in which the dashed line denotes the correct death rate at *δ* = 0.7).

Conversely, the two novel models (RQS: blue symbols and PWT: green symbols) accurately estimate the death rate even if many target cells survive. Note that it is not surprising that the RQS model provides better parameter estimates than the PWT model, because the artificial data was generated by the reduced standard model (Eqs. (1–3)), from which the RQS model was derived by a reasonable quasi-steady state assumption. The parameters are accurately estimated by all methods when target cells are severely depleted (i.e., when *f* approaches 0), reconfirming that the previous PWR model can accurately quantify the infected cell death when the target cells are severely depleted. This, for example, occurs in CXCR4-tropic simian–human immunodeficiency virus (SHIV) infections (which deplete naïve and memory CD4 T cells during the acute phase) [14,16,26-28]. In cases where target cells are not as strongly depleted, the new models do much better than the PWR model in estimating the death rate (Figure 3A), the viral replication rate (Figure 3B) and the basic reproduction number (Figure 3C). All models perform similarly on estimating the exponential growth rate (Figure 3D), because they all assume (implicitly or explicitly) that the number of target cells remains constant during the earliest phases of viral infections.

### Application to acute HIV-1 infection in a humanized mouse model

Having established that the novel models outperform the old one, they were fitted to the 21-day time courses of viral loads and target cells observed in five virus-infected humanized mice. From the parameters estimated with the individual mouse datasets (see Table 2 for the RQS model and Table 3 for the PWT model), we obtained the similar *g*_0_ ranging from 0.43 to 1.07 and from 0.48 to 1.06 per day in the RQS and PWT models, respectively. These estimates are not in disagreement with the replication rate of HIV-1 in human patients, which has been estimated to be 1.01 ± 0.37 per day [6]. We estimate a death rate of HIV-1 infected cells in humanized mice as *δ* ranging from 0.30 to 0.76 and from 0.38 to 0.76 per day with the RQS model and the PWT model, respectively (see Tables 2 and 3). Again, this result is in concordance with estimates of viral death rate in treated HIV-1 infected patients [10,11]. We then determined the basic reproduction number *R*_0_ of HIV-1 in humanized mice from the individual estimates of *T*(0), *r* and *δ*, obtaining *R*_0_ ranging from 1.58 to 3.88 and from 1.77 to 3.26 in the RQS and PWT models, respectively (see Tables 2 and 3). Since the mean of *R*_0_ corresponds to a predicted target cell nadir of *f* = 0.12 in the RQS model, the simple exponential model is not expected to perform equally well on this data (see Figure 3).

**Table 2:** Estimated parameter values and quantities derived from humanized mouse experiments by the novel RQS model

**ID**	***T*** **(0)**	***V*** **(0)**	***g***_**0**_	***r***	***β***	***δ***	***R***_**0**_
	**cells/ml**	**RNA copies/ml**	**day**^**-1**^	**(cell/ml)**^**-1**^・**day**^**-1**^**(×10**^**-6**^**)**	**(virion /ml)**^**-1**^・**day**^**-1**^**(×10**^**-6**^**)**	**day**^**-1**^	**---**
1	274139	2461	0.467	4.46	0.10	0.76	1.62
2	489096	652.1	1.065	3.37	0.03	0.58	2.83
3	93238.7	717.1	0.856	12.4	1.24	0.30	3.88
4	15847.5	1233	0.429	74.1	0.57	0.75	1.58
5	130580	1506	0.652	10.1	0.06	0.67	1.97
Mean	200580	1313	0.694	20.9	0.40	0.61	2.38

**Table 3:** Estimated parameter values and quantities derived from humanized mouse experiments by the novel PWT model

**ID**	***T*** **(0)**	***V*** **(0)**	***g***_**0**_	***r***	t^*^	**∆**	***δ***	***R***_**0**_
	**cells/ml**	**RNA copies/ml**	**day**^**-1**^	**(cell/ml)**^**-1**^・**day**^**-1**^**(×10**^**-6**^**)**	**day**	**day**^**-1**^	**day**^**-1**^	**---**
1	282809	2325	0.48	3.91	8.7	0.07	0.62	1.77
2	308636	589.7	1.06	5.86	5.8	0.13	0.75	2.42
3	79882.2	693.9	0.87	15.7	3.6	0.15	0.38	3.26
4	19465.0	1013	0.50	53.9	5.6	0.07	0.55	1.90
5	143159	774.8	0.87	11.4	5.1	0.08	0.76	2.14
Mean	166790	1079	0.76	18.2	5.8	0.10	0.61	2.30

**Figure 4:**
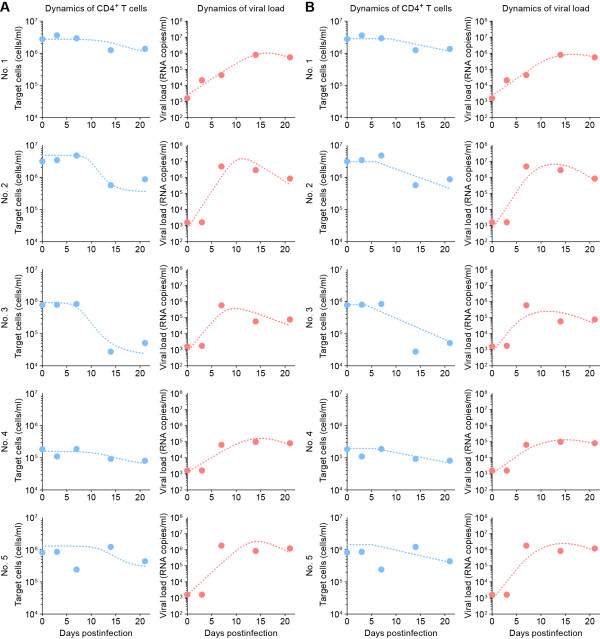
**Dynamics of HIV-1 infections in humanized mice.** The number of memory CD4^+^ T cells per ml of PB (left) and the viral RNA load per ml of plasma (light) in 5 humanized mice infected with HIV-1 are plotted as functions of time. The symbols denote experimental time course data and the dashed lines display the best fit of the RQS (Panel **(A)**) and PWT (Panel **(B)**) models to the data from each individual mouse.

The estimated parameter values of each individual mouse are given in Tables 2 and 3. Using the best-fit parameter estimates the behavior of RQS and PWT models is depicted together with the individual data in Figures 4A and B, respectively, which confirms that both models reasonably describe the acute phase of HIV-1 infection in humanized mice.

## Discussion

We here propose two novel models to quantify the most important parameters characterizing acute viral infections. Both models are major improvements over the previous simple exponential model [3,5-7] because that model has difficulties estimating the death rate of infected cells when target cells are not depleted after the viral load peak (see Figure 3). The novel models use the observed target cell densities when estimating the parameter values, and by using simulated data we have demonstrated that the new models typically outperform the previous model. Applying the new models to data obtained in humanized mice estimates that the rates at which the virus expands, and at which infected cells die, resemble those measured in humans.

The efficacy of vaccines eliciting cytotoxic immune responses [14-16] could be quantified by our new approach by comparing the estimated death rate of infected cells between normal and vaccinated individuals during acute infection. Additionally, by comparing the death rates estimated during acute infection with those estimated by perturbations with antiviral drugs during the chronic phase [4,10-13], we might be able to compare the impact of cellular immunity during both phases of an infection. Both examples illustrate that the new models can markedly improve our understanding of viral infection dynamics *in vivo*.

Our novel approach overcomes the difficulty the previous PWR model had with estimating the death rate of infected cells in situations where target are not severely depleted. Additionally, the choice between the two different models that we propose here can be made on the final densities of the target cells. If the number of target cells reaches a nadir during acute infection, the RQS model seems most appropriate. If the target cells continue to decrease the PWT model should be better.

Thus, whenever one has sufficient time course data from an acute infection, the new models should allow one to estimate the death rate of infected cells, and hence the *R*_0_, with reasonable accuracy. Indeed, in animal experiments using rhesus macaques [14-16,26-28], ferrets [30], and mice [17-20,31-34], both target cell densities and viral loads have been measured during the acute phase of viral infection. For example, the number of uninfected (and infected) cells in the lungs or respiratory tracts of macaques, ferrets and mice that were experimentally infected with influenza could be measured [30,31,34]. Using the CXCR4-tropic SHIV/macaque model, both target cell densities (naïve and memory CD4 T cells) and viral loads from PB have been measured [14,16,26-28]. The target cells of simian immunodeficiency virus (SIV), or CCR5-tropic SHIV infection (memory CD4 T cells expressing CCR5), have been measured from gastrointestinal mucosa samples [15]. Thus there are several infection models that can be analyzed with our new models.

## Conclusion

In this paper, we developed novel mathematical approaches to estimating parameters from acute viral infection data. We demonstrated that the new models outperform the previous model using simulated data. We quantified the dynamics of acute-phase HIV-1 infections by measuring their time course data in a humanized mouse model. Interestingly we find that the rates at which the virus expands, and at which infected cells die, are similar to those in humans.

## Competing interests

The authors declare that they have no competing interests.

## Authors’ contributions

Conceived and designed the study: HI, RDB, KA and SI. Analyzed the data: HI, SM and SI. Wrote the paper: HI, RDB, KA and SI. Contributed reagents/materials/analysis tools: KS, NM and YK. All authors read and approved the final manuscript.
